# 混合磨玻璃结节型肺腺癌脏层胸膜侵犯的临床特征和危险因素分析

**DOI:** 10.3779/j.issn.1009-3419.2022.102.07

**Published:** 2022-04-20

**Authors:** 程皓 符, 以恒 蒋, 佳云 葛, 梅 袁, 俊 王

**Affiliations:** 1 210000 南京，南京医科大学第一临床医学院 The First Clinical Medical College of Nanjing Medical University, Nanjing 210000, China; 2 210029 南京，南京医科大学第一附属医院影像科 Department of Radiology, The First Affiliated Hospital of Nanjing Medical University, Nanjing 210029, China; 3 210029 南京，南京医科大学第一附属医院胸外科 Department of Thoracic Surgery, The First Affiliated Hospital of Nanjing Medical University, Nanjing 210029, China

**Keywords:** 混合性磨玻璃结节, 肺肿瘤, 胸膜侵犯, 计算机断层扫描, Mixed ground glass nodule, Lung neoplasms, Pleural invasion, Computed tomography

## Abstract

**背景与目的:**

目前国内肺癌仍是发病率和致死率最高的恶性肿瘤，肺腺癌是其最常见的亚型，影像学中表现为混合性磨玻璃结节(mixed ground glass nodule, mGGN)的肺癌逐渐增多。脏层胸膜侵犯(visceral pleural invasion, VPI)是影响mGGN型肺腺癌预后的重要因素。本研究旨在探索分析mGGN型肺腺癌发生VPI的危险因素。

**方法:**

回顾性分析2016年11月-2019年11月南京医科大学第一附属医院收治的接受根治性手术的128例mGGN型肺腺癌患者的临床资料，包括影像、病理和生物学特征，其中男性40例、女性88例，年龄30岁-81(60.3±9.3)岁。采用单因素卡方检验与多因素*Logistic*回归分析调查mGGN型肺腺癌发生VPI的各项危险因素。

**结果:**

符合纳入标准的128例mGGN型肺腺癌患者中57例被诊断有VPI发生。性别、实性成分最大径、实性成分比例(consolidation tumor ratio, CTR)、毛刺征、肺部疾病史、高血压家族史、肿瘤-胸膜空间位置分型(relation of lesion to pleura, RLP)、支气管与结节走行关系在VPI(+)组和VPI(-)组间的差异均有统计学意义(*P* < 0.05)。*Logistic*多因素回归分析发现RLP(OR=3.529, 95%CI: 1.430-8.713, *P*=0.006)和支气管与结节走行关系(OR=3.993, 95%CI: 1.517-10.51, *P*=0.005)是VPI发生的独立危险因素(*P* < 0.05)。

**结论:**

临床诊治应综合上述参数评估mGGN型肺腺癌发生VPI的可能性。RLP和支气管与结节走行关系异常作为VPI的独立危险因素，对判断mGGN型肺腺癌发生VPI具有一定的指导意义。

## Abstract (en)

**Background and objective:**

Lung cancer is still the malignant tumor with the highest morbidity and mortality in China. Lung adenocarcinoma is the most common subtype, and the number of lung cancer presenting as mixed ground glass nodule (mGGN) in imaging is gradually increasing. Visceral pleural invasion (VPI) is an important factor affecting the prognosis of mGGN type lung adenocarcinoma. The aim of the study is to explore and analyze the risk factors for VPI in mGGN type lung adenocarcinoma.

**Methods:**

From November 2016 to November 2019, 128 patients with mGGN lung adenocarcinoma underwent radical surgical resection in the First Affiliated Hospital of Nanjing Medical University. Their clinical data, including imaging, pathological and biological features, were collected and analyzed retrospectively. There were 40 males and 88 females, aged 60.3±9.3 years ranging from 30 to 81 years. Single factor Chi-square test and multivariate *Logistic* regression were used to analyze the risk factors of VPI in mGGN type lung adenocarcinoma.

**Results:**

Among 128 mGGN patients who met the inclusion criteria, 57 cases were pathologically confirmed with pleural invasion. Between the VPI (+) and VPI (-) group (*P* < 0.05), there were significant differences in gender, maximum diameter of solid component, consolidation tumor ratio (CTR), spicule sign, history of lung disease, family history of hypertension, relation of lesion to pleura (RLP), coursing relationship between bronchi and nodules. In multivariate *Logistic* regression analysis, RLP (OR=3.529, 95%CI: 1.430-8.713, *P*=0.006) and coursing relationship between bronchi and nodules (OR=3.993, 95%CI: 1.517-10.51, *P*=0.005) were found to be independent risk factors for VPI (*P* < 0.05).

**Conclusion:**

The possibility of VPI in m GGN lung adenocarcinoma should be evaluated by combining these parameters in clinical diagnosis and treatment. As independent risk factors, RLP and coursing relationship between bronchi and nodules are instructive to identify VPI in mGGN type lung adenocarcinoma.

非小细胞肺癌(non-small cell lung cancer, NSCLC)是世界范围内死亡率很高的恶性肿瘤，对人类健康以及公共卫生提出了巨大挑战^[1, 2]^。NSCLC包括腺癌、鳞状细胞癌和大细胞癌在内的多种分型，其中肺腺癌发病占比最大。近年来，随着影像低剂量计算机断层扫描(low-dose computed tomography, LDCT)不断发展并在临床广泛应用，肺部磨玻璃结节(ground glass nodule, GGN)检出率不断增高，在炎症、肿瘤、痰栓或者肺泡腔内出血等情况下均可出现肺部GGN表现。与纯GGN(pure GGN, pGGN)相比，混合GGN(mixed GGN, mGGN)在CT图像上表现为含有部分实性成分的模糊影。已有学者研究^[3]^证实，mGGN恶性概率较pGGN高。因此，影像学表现为mGGN的肺腺癌逐步成为临床研究的重点。手术完整切除病灶仍是目前早期mGGN型肺腺癌最主要的治疗手段^[4]^，但即使是早期肺癌也存在术后复发的情况^[5]^。有研究^[6]^报道称脏层胸膜侵犯(visceral pleural invasion, VPI)是mGGN型肺腺癌预后不佳的高风险因素之一。而国际肺癌研究协会(International Association for the Study of Lung Cancer, IASLC)在肺癌第八版肿瘤原发灶-淋巴结-转移(tumor-node metastasis, TNM)分期标准中指出，将直径≤3 cm、合并VPI的早期NSCLC患者由Ia期(T1N0M0)升至Ib期(T2aN0M0)。由此可见，VPI作为危险因素直接影响早期肺癌患者的分期和预后^[7]^。在精准医疗时代，术前分析预测VPI的发生对mGGN型肺腺癌患者治疗方案的制定、优化以及预后判断具有指导意义。目前国内外对于mGGN型肺腺癌VPI预测的报道仍然较少，尚有待深入研究。本研究回顾性分析mGGN型肺腺癌患者的临床资料，包括影像、病理和生物学特征，探讨mGGN型肺腺癌发生VPI的相关高危因素，以期提高肺腺癌患者VPI术前预测的准确性，为拟定诊治方案提供参考依据。

## 1 资料与方法

### 1.1 一般资料

收集南京医科大学第一附属医院2016年11月-2019年11月行手术切除的2, 246例肺部mGGN患者的病史资料。按照年龄、性别、吸烟史、病变大小等匹配后初筛出1, 150例肺腺癌患者，考虑到阴性、阳性数据集匹配性，进一步筛除纳入病例，最终128例患者符合纳入标准。

### 1.2 纳入与排除标准

纳入标准：①肺部mGGN(最大直径≤3 cm); ②经病理学检查确诊为肺腺癌; ③已获得患者及其家属知情同意。排除标准：①病灶直径 > 3 cm; ②病理检查病理结果显示良性或其他亚型病变; ③临床资料或影像学资料不完整; ④存在淋巴结转移或远处转移; ⑤合并脉管及支气管侵犯。

### 1.3 纳入信息

纳入的临床信息包括：性别、年龄、吸烟史、肺部疾病史(如肺结核、肺部结节、肺部感染等)、其他恶性肿瘤史(如子宫肌瘤、胃间质肿瘤、乳腺癌、食管癌等)、高血压家族史以及肿瘤家族史。

该研究获得了南京医科大学第一附属医院伦理委员会的批准，伦理审查编号：2016-SR-102、2019-SR-450、2019-SR-451、2020-SRFA-005。

### 1.4 研究方法

#### 1.4.1 影像学检查方法

CT扫描与图像重建：使用西门子128层螺旋CT扫描(Siemens, Definition AS+; Malvern, Pa)，层厚1.5 mm，层间距1.2 mm。参数设置：电压100 kVp或者120 kVp，mAs设置应用CARE Dose4D个体化选择，扫描矩阵512×512。窗宽和窗位：肺窗(窗宽：1, 200 HU; 窗位：-600 HU)，纵隔窗(窗宽：350 HU; 窗位：50 HU)。

采用双盲法，由2名具有10年以上影像诊断工作经验的胸部影像诊断医师分别对CT图像进行评估。两人诊断意见不统一时，经协商解决意见分歧。收集2名医师的诊断结果并分析观察者所测数据的一致性(κ)。首先对VPI进行定性判定(图 1)。

**图 1:**
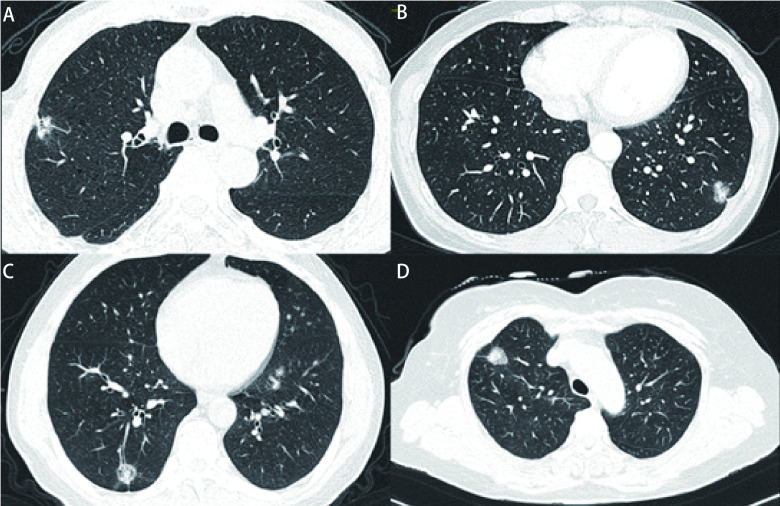
不同亚型肺腺癌VP(+)与(-)的典型影像学表现。A：典型肺腺癌(腺泡型)伴VPI(+)影像学表现; B：典型肺腺癌(贴壁型)伴VPI(+)影像学表现; C：典型肺腺癌(腺泡型)伴VPI(-)影像学表现; D：典型肺腺癌(贴壁型)伴VPI(-)影像学表现。 Typical imaging features in different subtypes of lung adenocarcinoma with VPI (+) or VPI (-). A: Imaging features of typical lung adenocarcinoma (acinar type) with VPI (+); B: Imaging features of typical lung adenocarcinoma (adherent type) with VPI (+); C: Imaging features of typical lung adenocarcinoma (acinar type) with VPI (-); D: Imaging features of typical lung adenocarcinoma (adherent type) with VPI (-). VPI: visceral pleural invasion.

确定VPI存在与否之后，拟定测定分析的CT表现包括：病变位置、结节大小、结节性质(实性结节、部分实性结节或GGN)、实性成分最大径、实性成分比例(consolidation tumor ratio, CTR)、分叶征、毛刺征、空洞、蜂窝征、血管穿行征、肿瘤-胸膜空间位置分型(relation of lesion to pleura, RLP)和支气管与结节走行关系。最后两个影像学表现涉及多种类型。

RLP可分为5种类型：1型：病变和胸膜之间不存在接触; 2型：病灶表面和胸膜表面存在线状连续，且胸膜无皱缩; 3型：病灶与胸膜紧邻，且胸膜无皱缩; 4型：病灶表面和胸膜表面存在线状连续，且胸膜有皱缩; 5型：病灶与胸膜间基底接触较宽(接触面长度 > 结节最大径的50%)，且胸膜有皱缩。其中，4型和5型代表胸膜凹陷征阳性组，其余划归为阴性组^[8]^。RLP 5种类型分别用RLP 1型-RLP 5型表述。

支气管与结节走行关系可分为6种类型：1型：无穿行支气管; 2型：支气管狭窄截断; 3型：实性部分支气管穿行伴扭曲或不伴扭曲扩张; 4型：在磨玻璃部分穿行并伴有扭曲扩张未进入实性部分; 5型：在磨玻璃成分中正常穿行; 6型：支气管在结节旁穿行。其中，5型和6型代表支气管与结节正常走行关系，其余划归为异常组。必须单独说明的是，1型无穿行支气管在一般情况下常见于肺部无异常疾病的患者，但是本次研究纳入患者皆为肺腺癌患者，出现1型有很大程度是因为穿行支气管狭窄截断(同2型)的同时影像学未完整记录，故1型亦纳入异常组。

#### 1.4.2 病理学检测方法

经穿刺或手术活检获取病理标本，由1名有10年工作经验的病理科医生参与判断。若苏木精-伊红(hematoxylin-eosin, HE)染色无法准确区分有无胸膜侵犯，进行弹力纤维染色以判断有无VPI。弹力纤维染色的步骤：①脱蜡至水; ②切片置于Verhoeff染液中，浸染20 min; ③自来水快速冲洗; ④经Verhoeff分化液处理，弹力纤维呈分化为黑色，背景为灰色; ⑤自来水快速冲洗; ⑥95%乙醇快速冲洗脱碘。⑦使用Van Gieson染色液或伊红复染30 s，吸干多余染液; ⑧无水乙醇快速脱水，中性树胶封固。根据IASLC肺癌第八版TNM分期方案：PL0指胸膜侵犯未累及弹性层，PL1指病变侵犯超过脏层胸膜弹力层，PL2指侵袭脏层胸膜表面。本研究将PL1和PL2归为VPI(+)组。

依据病理学检测结果将样本分为贴壁型、乳头型、微乳头型、实性型和腺泡型5种亚型。既往关于病理特征、病灶生长模式和预后生存分析^[9]^的研究表明，各亚型之间存在侵袭性差异，综合评价肺腺癌亚型侵袭性的相似程度，依据侵袭性从低到高将其划分为I、II、III三组。I组：贴壁型和腺泡型; II组：乳头型; III组：微乳头型和实体型肺腺癌。

#### 1.4.3 免疫组学检测方法

癌胚抗原(carcinoembryonic antigen, CEA)、细胞角蛋白19片段抗原(cytokeratin 19 fragment antigen 21-1, CYFRA21-1)与神经元特异性烯醇化酶(neuron-specific enolase, NSE)的测定是通过静脉取外周血血清，使用罗氏诊断公司(Roche Diagnostics GmbH, Mannheim, Germany)的测定试剂盒，采用电化学发光法定量检测CEA、NSE、CYFRA21-1水平。其中CEA的正常值为0 ng/mL-4.7 ng/mL，NSE的正常值为0 mg/mL-16.3 mg/mL，CYFRA21-1的正常值为0 ng/mL-3.30 ng/mL，测定值超过正常值范围则该项异常。

Ki-67的测定是通过将经手术获取的病理标本以10%甲醛溶液固定，常规行石蜡包埋。将采集到的组织标本切成4 μm厚的薄片，PBS缓冲液冲洗后，用柠檬酸缓冲液修复组织标本，滴加过氧化氢溶液，室温下孵育10 min后，加入Ki-67抗体在室温下继续孵育30 min，PBS液冲洗后采用DAB显色，再用HE染色，置于电子光镜下观察，若细胞核内有棕黄色颗粒物，即可判定为细胞Ki-67染色。在上述Ki-67细胞染色最强的区域进行计数，当1, 000个肿瘤细胞中≥20%的细胞被染色时，则判定该样本对应的患者为Ki-67阳性。

#### 1.4.4 基因分子检测方法

表皮生长因子受体(epidermal growth factor receptor, *EGFR*)突变信息由二代测序(next-generation sequencing, NGS)方法获得(广州燃石医学检验所有限公司，测序仪llumina MiSeqDx平台)。

### 1.5 统计学方法

采用Stata 14软件进行数据分析。在单因素分析中，对非连续性变量采用卡方检验，对连续性变量采用双样本*t*检验。同时将其中*P* < 0.1的因素纳入二元*Logistic*多因素回归分析，*P* < 0.05为差异具有统计学意义。使用κ统计评估观察者间的一致性。

## 2 结果

### 2.1 基础信息

128例符合纳入标准的肺腺癌mGGN患者，年龄30岁-81岁，平均年龄(60.3±9.3)岁; VPI(+)57例，VPI(-)71例，阳性率为44.5%; VPI(+)患者中男性24例，女性33例。本实验两位观察者间有高度的一致性(κ=0.737, *P* < 0.001)。

### 2.2 单因素分析结果

对128例患者临床资料进行单因素分析，结果发现患者性别(*P*=0.018)、高血压家族史(*P*=0.031)在VPI(+)组和VPI(-)组间的差异具有统计学意义(*P* < 0.05)，而年龄、吸烟史、肺部疾病史、其他恶性肿瘤病史和肿瘤家族史在VPI(+)组和VPI(-)组无统计学差异(*P* > 0.05)。mGGN在肺部病变的位置在VPI(+)组和VPI(-)组也无统计学差异，而且病理及其组织学亚型单因素分析结果显示，低侵袭性肺腺癌(I组：贴壁型和腺泡型)、中侵袭性肺腺癌(II组：乳头型)和高侵袭性肺腺癌(III组：微乳头型和实体型)在VPI(+)组和VPI(-)组不存在统计学差异(*P* > 0.05)(表 1)。

**表 1:** 128例肺腺癌混合磨玻璃结节患者的临床资料单因素分析 Univariate analysis of clinical variables of 128 patients with mixed ground glass nodules of lung adenocarcinoma

Variables	*n*	VPI		*t*/*χ*^*2*^	*P*
		(-)	(+)		
Gender^a^				5.636	0.018
Male	40	16	24		
Female	88	55	33		
Age (Mean±SD, yr)^b^	60.3±9.3	61.2±9.4	59.1±9.1	1.273	0.205
Location of lesion^a^				3.457	0.485
LUL^a^	32	14	18	2.372	0.124
LLL^a^	16	8	8	0.221	0.638
RUL^a^	47	28	19	0.507	0.477
RML^a^	13	9	4	1.109	0.292
RLL^a^	20	12	8	0.197	0.657
Pathology and histological subtypes^a^				2.332	0.312
Ⅰ lepidic predominant/acinar^a^	74	43	31	0.495	0.482
Ⅱ papillary^a^	36	21	15	0.166	0.683
Ⅲ micropapillary/solida	18	7	11	2.331	0.127
Smoking history^a^				0.533	0.465
With	15	7	8		
Without	113	64	49		
History of lung diseases^c^				1.951	0.081
With	8	7	2		
Without	120	77	65		
History of other malignant tumor diseases^a^				0.097	0.755
With	21	11	10		
Without	107	60	47		
Family history of hypertension^c^				4.658	0.031
With	3	3	0		
Without	125	68	57		
Family history of carcinoma^c^				0.002	0.963
With	8	4	4		
Without	120	67	53		
^a^: *Pearson* *χ*^*2*^ test conducted; ^b^: Student *t* test conducted; ^c^: *Yates*’s correction for continuity conducted. LUL: left upper lobe; LLL: left lower lobe; RUL: right upper lobe; RML: right middle lobe; RLL: right lower lobe.					

我们进一步分析患者外周血CEA、CYFRA21-1、NSE水平、Ki-67免疫组化和*EGFR*突变基因状态，结果同样也未出现统计学差异(表 2)。而在CT影像特征的单因素分析中，结果显示实性成分最大径(*P* < 0.001)、CTR(*P* < 0.001)、RLP(*P* < 0.001)、毛刺征(*P*=0.003)和支气管与结节走行关系(*P* < 0.001)在VPI(+)组和VPI(-)组间的差异具有统计学意义。轴位最大径、分叶征、空洞征、蜂窝征、血管集束征各型间比较与总体数据分析结果均无显著统计学差异(*P* > 0.05)(表 3)。

**表 2:** 128例肺腺癌混合磨玻璃结节患者的免疫组学与基因突变单因素分析 Univariate analysis of immunohistochemistrical and genetic variables of 128 patients with mixed ground glass nodules of lung adenocarcinoma

Variables	*n*	VPI		*χ*^*2*^	*P*
		(-)	(+)		
CEA^a^				0.001	0.971
Normal	99	55	44		
Higher than normal	29	16	13		
CYFRA21-1^a^				1.048	0.306
Normal	86	45	41		
Higher than normal	42	26	16		
NSE^a^				0.004	0.951
Normal	58	32	26		
Higher than normal	70	39	31		
Ki-67^a^				0.254	0.615
< 20%	110	62	48		
≥20%	18	9	9		
*EGFR* mutation^a^				0.750	0.387
With	75	44	31		
Without	53	27	26		
^a^: *Pearson* *χ*^*2*^ test conducted. CEA: carcinoembryonic antigen; CYFRA21-1: cytokeratin 19 fragment antigen 21-1; NSE: neuron-specific enolase; EGFR: epidermal growth factor receptor.					

**表 3:** 128例肺腺癌混合磨玻璃结节患者的CT影像特征单因素分析 Univariate analysis of CT imaging features of 128 patients with mixed ground glass nodules of lung adenocarcinoma

Variables	*n*	VPI		*t*/*χ*^*2*^	*P*
		(-)	(+)		
Maximum shaft diameter (Mean±SD, mm)^a^	18.3±5.1	18.4±5.5	18.2±4.4	0.312	0.756
Maximum diameter of solid component (Mean±SD, mm)^a^	9.3±4.9	7.7±4.1	11.4±5.1	-4.635	< 0.001
CTR (Mean±SD)^a^	0.5±0.2	0.4±0.2	0.6±0.3	-5.023	< 0.001
Relation of lesion to pleura (RLP)^a^				23.844	< 0.001
Without pleural indentation	73	52	21	17.092	< 0.001
Ⅰ irrelevant^a^	38	32	6	18.074	< 0.001
Ⅱ linear tractive^a^	22	14	8	0.718	0.397
Ⅲ appressed^a^	13	6	7	0.508	0.476
With pleural indentation	55	19	36		
Ⅳ linear tractive with pleural indentation^a^	32	10	22	10.132	0.001
Ⅴ wide basal pleural indentation^a^	23	9	14	3.030	0.082
Lobulation sign^a^				1.419	0.492
Without^a^	100	54	46	0.399	0.522
Slight lobulation^a^	25	16	9	0.915	0.339
Deep lobulation^b^	3	1	2	0.037	0.847
Spicule sign^a^				8.654	0.003
Without	113	68	45		
With	15	3	12		
Intrapulmonary air containing space^a^				2.533	0.282
Without^a^	117	66	51	0.489	0.485
With one^b^	9	5	4	0.125	0.724
With two and above^b^	2	0	2	0.764	0.382
Honeycomb sign^b^				0.001	0.975
Without	118	66	52		
With	10	5	5		
Coursing relationship between bronchus and nodule^a^				11.861	0.001
Normal	48	36	12		
Abnormal	80	35	45		
Vascular convergence sign^b^				1.170	0.279
Without	121	69	52		
With	7	2	5		
^a^: *Pearson* *χ*^*2*^ test conducted; ^b^: *Yates’s* correction for continuity conducted; CT: computed tomography; CTR: consolidation tumor ratio; RLP: relation of lesion to pleura.					

按照病例的RLP分型进行分析，结果显示相对于胸膜凹陷征阴性组，胸膜凹陷征阳性组更易发生VPI(36/55, 65.5% *vs* 21/73, 28.8%)，具有统计学差异(*P*=0.001); 各单项RLP分型间研究显示，RLP 4型[VPI(+)组22例，VPI(-)组10例，*P*=0.001]对于VPI的发生具有更大的提示意义(表 3)。

### 2.3 独立危险因素分析结果

将以上*P* < 0.1的因素纳入*Logistic*多因素回归分析中，发现支气管与结节走行关系和RLP是VPI发生的独立危险因素(*P* < 0.05)。而性别、实性成分最大径、CTR与毛刺征均不是发生VPI的独立危险因素(表 4)。

**表 4:** 128例肺腺癌混合磨玻璃结节患者发生VPI的*Logistic*回归分析 *Logistic* regression analysis of VPI in 128 patients with mixed ground glass nodules of lung adenocarcinoma

Variables	Coef.	Std. Err.	*P*	OR	OR (95%CI)
Gender	0.837	0.477	0.079	2.310	0.907-5.885
CTR	1.914	1.593	0.230	6.780	0.299-153.810
Pleural indentation	1.261	0.461	0.006	3.529	1.430-8.713
Maximum diameter of solid component	0.053	0.079	0.499	1.055	0.904-1.231
Spicule sign	0.479	0.793	0.546	1.615	0.341-7.639
Coursing relationship between bronchi and nodules	1.385	0.494	0.005	3.993	1.517-10.506
Constant	-3.493	0.717	0.000	0.030	0.007-0.124
Coef.: regression coefficient; Std. Err.: standard error; OR: odd ratio; CI: confidence interval.					

## 3 讨论

VPI是影响mGGN型肺腺癌转归的重要征象之一。有研究^[10]^指出，VPI(+)代表肺腺癌恶性程度增高，而合并VPI的NSCLC患者往往预后不佳。目前VPI的诊断主要依赖于活检或者手术标本的病理弹力纤维染色检测，这些方法需要在术后等待较长的时间，无法在术中快速病理中实现确诊，无法指导手术的即时决策^[11]^。鉴于VPI在mGGN型肺腺癌精准诊治中的重要地位，通过对影像学显示mGGN的病灶进行VPI的预测，在一定程度上可以反映病变的严重程度以及患者预后情况，为精确诊断、制定治疗方案和判定预后提供指导依据。既往有多位学者^[12, 13]^研究了肺腺癌各类特征与VPI的关系。Qi等^[14]^使用多变量分析来评估VPI的危险因素，显示性别、肿瘤的密度以及胸膜凹陷征等因素与VPI有关。Manac'h等^[15]^的研究结果也显示肿瘤的最大径与VPI的发生有统计学关联。但以往研究多数着眼于实体性肺腺癌，并没有细化到mGGN型肺腺癌，很少有关于mGGN型肺腺癌发生VPI的独立危险因素的分析。此外，外周血清免疫化合物与基因突变检测在VPI诊断中的探索也少有报道。

本研究首次纳入128例mGGN型肺腺癌患者的影像学资料，同时纳入患者的临床病理信息、免疫组化、基因突变的信息资料，探索患者各类特征和发生VPI之间的关系。经单因素分析筛选出相关危险因素后，再使用*Logistic*多因素回归分析，进一步明确mGGN型肺腺癌发生VPI的独立危险因素，建立全新的临床病理-影像-生物学模式，综合判断mGGN型肺腺癌中VPI发生的可能性。目前，对于GGN型肺腺癌的影像学表现，已有诸多研究显示：RLP^[16]^、毛刺征^[17]^和支气管穿行征^[17]^等特征性影像学表现均可为判断肺部小结节良、恶性和浸润程度提供一定的依据。本研究结果显示上述征象与mGGN型肺腺癌的VPI发生同样存在相关性，并进一步得出结论：其中具有明显意义的是RLP和支气管与结节走行关系。

RLP分型是依据肿瘤与胸膜之间的阴影关系得出的空间位置关系，其中4型、5型合称胸膜凹陷征，产生与肿瘤体内的瘢痕组织牵拉临近的脏层胸膜组织有关。既往多项报道^[16, 18]^显示胸膜凹陷征是肺腺癌发生VPI的重要危险因素之一，在本研究中也验证了以上结论(*P* < 0.001)。将RLP分型进一步细化并分析数据，表明合称胸膜凹陷征的2个亚型RLP 4型、RLP 5型(15/50, 30.0%)VPI发生率较RLP 1型、RLP 2型和RLP 3型(5/118, 4.24%)高。该结果说明了有胸膜凹陷征的患者更倾向于发生VPI，这与既往的研究结果^[16]^一致，有利于在临床影像和术中辨认VPI; 值得注意的是，本研究结果显示6例RLP 1型患者发生VPI，提示隐匿型VPI的存在，判断时需进一步核实。进一步*Logistic*多因素回归分析显示，RLP亦是一个独立的危险因素(OR=5.317, 95%CI: 1.880-15.036, *P*=0.002)。有趣的是，纳入研究的55例胸膜凹陷征病例(32例RLP 4型、23例RLP 5型)中尚有19例显示VPI(-)，提示了胸膜凹陷征与VPI并不是绝对共存关系。Gallagher等^[19]^认为胸膜凹陷征的发生与成纤维细胞的增生和炎症浸润相关，即胸膜凹陷征仅表示纤维成分牵拉胸膜，不一定等同于VPI，其数据也与本研究病例数据分布相似。

罗继元等^[20]^研究发现不同恶性程度的肺腺癌，其支气管与结节走行关系存在差异，这间接揭示了支气管与结节走行关系与VPI的潜在关联。本研究的单因素卡方检验提示支气管与结节走行关系是VPI发生的重要影响因素。进行多因素*Logistic*回归分析对比分析后，发现支气管与结节走行关系是一个独立危险因素，异常走行关系对于VPI的发生以及预后具有不可忽视的影响，在临床应该着重注意截断、扭曲、扩张等特殊走行征象，这也与高益萍等^[21]^研究结果相符。在本研究中，分叶征不具有统计学意义，这与彭兆晖等^[22]^研究不符，有待将mGGN型和实性型肺腺癌对比分析，对该结论进行进一步的探讨。

目前已有报道^[23]^称，肺部结节内CTR越高，其诊断为恶性的可能性越高，发生淋巴结转移和术后复发的概率也越大。本研究结果也提示CTR在VPI(+)和VPI(-)呈现统计学差异，也间接反映了实性成分和预后的相关性。有趣的是，Ye等^[24]^研究结果提示部分实性结节与实性结节临床病理特征存在差异，肿瘤CTR、实性成分大小和结节大小均不能预测部分实性结节患者的预后，但该研究中未纳入VPI信息，故仅具有一定参考价值。

本研究还探讨了病理亚型对VPI的影响。根据侵袭性将肺腺癌分为低、中、高三组，我们发现高侵袭性肺腺癌发生VPI的概率(61.1%)高于中、低侵袭性肺腺癌(41.8%)，虽然统计学差异不显著，但这与Shimizu等^[25]^的研究结果相符，提示了病理亚型对于VPI预测的潜在价值。此外，本研究结果显示，性别对VPI发生的影响具有统计学差异，而年龄、病灶位置、吸烟史等因素无统计学差异(*P* > 0.05)，这与Qi^[14]^、Bellier^[26]^、Kobayashi等^[27]^学者的研究结果不完全相符，笔者推测可能与样本的选择偏倚有关; 研究中发现高血压家族史与VPI间存在统计学意义，关于高血压家族史在VPI发生中的作用，是否与既往报道的肿瘤相关代谢综合征^[28]^有关尚有待进一步研究。

就我国当前医疗水平来说，对于特征显著的肺腺癌患者的诊断难度相对较小，但早期患者或者需要长期观察的患者，如RLP 1型患者，其各类特征缺乏特异性，必须结合其他手段综合评估其发生VPI的可能性。因此，综合多学科检测的诊断方法具有广泛前景。鲍彤等^[29]^发现，血清CEA和CYFRA21-1与影像学特征模型的结合，提高了预测孤立性肺结节恶性发生概率的准确性。Le等^[30]^的研究指出，*EGFR*突变型的周围型肺腺癌可通过调控microRNA-135b的过表达，提高肿瘤组织侵犯胸膜的能力。然而在本研究内，数项免疫组学指标和基因突变比率未提示统计学差异，期待在后续研究中通过可视化的影像特征和分子组学特征共同提高非特异病灶的高风险因素预测能力。

本研究存在一些局限。首先，本研究属于单中心研究，样本量相对不足，缺乏外部验证; 其次，回顾性研究可能导致病例搜集存在选择性偏倚; 最后，由于各种因素的干扰，未能对患者进行长期有效的随访。此外，VPI患者的手术方式和生存数据未能纳入分析。我们期待多中心、前瞻性的研究对本研究结果进行验证与拓展。

综上，在最大径≤3 cm的mGGN型肺腺癌中，RLP、支气管与结节走行关系是判断VPI的重要危险因素。影像-病理-生物学综合模型可更好地预测评估mGGN型肺腺癌VPI，为优化治疗方案以及判断预后等方面提供指导意义。
